# A Correction

**Published:** 1888-11

**Authors:** 


"A CORRECTION.—The *Boston Medical and Surgical Journal*, from which an extract was quoted by *The Medical Record* bearing on the composition of several artificial foods, publishes a correction based upon the analyses of Professors Edwin Waller and A. A. Breneman regarding Reed & Carnrick's soluble food, to the effect that 38.26 per cent. of the albuminoids which it contains, are in soluble form, that no 'hard unchanged particles of casein' were found, that the casein is partially rendered soluble by the action of the digestive ferment. That the proportion of the albuminoids in Liquid Peptonoids is limited only by the quantity which can be kept unchanged in solution, that sixteen per cent. of alcohol is necessary to prevent decomposition of the albuminoids, and that no greater than three per cent. of these can be held in solution in this liquid. We publish the correction from the same source as the original quotation as an act of justice to all concerned, regretting that we, in common with our Boston contemporary, were in any manner misled by what appeared to be a well-authenticated official report."—*Med. Record, Oct. 13, 1888.*

---

DR. JOS. HOLT, ex-President Louisiana State Board of Health, who made so much fame by the invention and execution of his plan of "maritime sanitation," removed to Portland, Oregon, sometime since. We are pleased to learn that he has been tendered, and has accepted, the chair of obstetrics in the Oregon Medical College.

